# Targeting the NLRP3 Inflammasome with Colchicine in COVID-19: Therapeutic Evidence and Future Implications for Influenza

**DOI:** 10.3390/ijms27156827

**Published:** 2026-07-30

**Authors:** Vanyo Mitev

**Affiliations:** 1 Research Institute of Innovative Medical Science, Medical University Sofia, Akad. Ivan Geshov 15, 1431 Sofia, Bulgaria; vmitev@mu-sofia.bg; 2 Department of Medical Chemistry and Biochemistry, Medical Faculty, Medical University Sofia, Akad. Ivan Geshov 15, 1431 Sofia, Bulgaria

**Keywords:** NLRP3 inflammasome, COVID-19, infuenza, colchicine, cytokine storm, immunothrombosis

## Abstract

Coronavirus disease 2019 (COVID-19) and influenza share key pathogenic mechanisms, including viral invasion, dysregulated innate immune activation, and the development of severe systemic complications. A central mediator of disease progression in both infections is the hyperactivation of the NLRP3 inflammasome, which drives excessive production of pro-inflammatory cytokines, immunothrombosis, multiorgan injury, and increased mortality. Colchicine possesses a unique pharmacokinetic property of preferential accumulation within myeloid cells, where sufficiently high intracellular concentrations inhibit NLRP3 inflammasome activation. This mechanism provides a biological rationale for preventing the cytokine storm and its downstream consequences when colchicine is administered early during infection. Available pharmacokinetic, toxicological, and clinical evidence suggests that loading doses of colchicine up to approximately 0.05 mg/kg body weight can be administered safely in appropriately selected patients, provided that clinically significant drug–drug interactions and hepatic or renal impairment are carefully excluded. The widely accepted belief that total doses of 7–7.5 mg are inherently lethal appears to reflect historical cases complicated by drug interactions and/or hepatic or renal impairment, rather than toxicity attributable to colchicine dose alone. These observations support reconsideration of current guideline recommendations regarding colchicine dosing. In particular, cumulative doses below 0.1 mg/kg appear to be consistently safe, whereas doses between 0.1 and 0.2 mg/kg are associated with only a low risk of toxicity and rarely with severe intoxication. Reassessment of colchicine dosing strategies may therefore be warranted to optimize NLRP3 inflammasome inhibition and improve outcomes in patients with COVID-19 and influenza.

## 1. Introduction

The COVID-19 pandemic has resulted in profound global health, economic, and societal consequences. Beyond the millions of deaths attributed directly or indirectly to SARS-CoV-2 infection, a substantial proportion of survivors have experienced persistent symptoms and long-term health complications collectively referred to as Long COVID. The pandemic has also adversely affected mental health, education, social cohesion, economic development, and political systems [1,2].

COVID-19 has become the most intensively studied disease in medical history. By 2024, more than two million researchers had contributed to the COVID-19 scientific literature, generating over ten million citations. This unprecedented scientific response has produced an enormous body of evidence spanning virology, epidemiology, therapeutics, vaccines, public health interventions, and the broader socioeconomic consequences of the pandemic [3,4].

Therapeutic research has been particularly extensive. Several hundred drugs have been evaluated in clinical studies for COVID-19, while thousands of compounds have undergone preclinical investigation. By 2025–2026, major clinical trial registries and evidence databases listed more than 12,000 registered COVID-19-related clinical trials. Despite this extraordinary research effort and the substantial financial resources invested, relatively few pharmacological interventions have demonstrated clear and consistent clinical benefits. Many initially promising therapeutic approaches ultimately failed to confirm efficacy in large randomized controlled trials [5].

This discrepancy between the unprecedented scale of scientific activity and the limited number of highly effective therapeutic options highlights both the complexity of COVID-19 pathophysiology and the challenges of translating preliminary findings into robust clinical evidence.

Despite the unprecedented volume of research, only a limited number of interventions have received recommendations from the World Health Organization (WHO) for the prevention or treatment of COVID-19. These include vaccines; antiviral agents such as nirmatrelvir/ritonavir (Paxlovid), remdesivir, and molnupiravir; anti-inflammatory treatment with dexamethasone; immunomodulatory agents including tocilizumab and baricitinib; anticoagulation with heparin in appropriately selected hospitalized patients; and optimized supportive care, particularly oxygen therapy and intensive care management [6].

Among these interventions, systemic corticosteroids—most notably dexamethasone—have emerged as one of the most consistently supported treatments for severe and critical COVID-19. Randomized clinical trials and subsequent meta-analyses have demonstrated significant reductions in mortality among patients requiring supplemental oxygen or respiratory support, with relative risk reductions generally estimated at approximately 20–35%. These findings established dexamethasone as a cornerstone of treatment for severe COVID-19 and represent one of the most important therapeutic advances achieved during the pandemic [7].

Vaccines provide substantial protection against severe COVID-19, hospitalization, and death. Their effectiveness in preventing SARS-CoV-2 infection, however, is only partial and varies according to circulating variants, prior immunity, and time since vaccination. The World Health Organization continues to emphasize that vaccinated individuals who develop COVID-19 generally experience a milder disease course and a lower risk of severe outcomes [8].

Evidence regarding the effectiveness of antiviral therapies remains heterogeneous. The clinical value of nirmatrelvir/ritonavir (Paxlovid), the WHO’s preferred antiviral agents, has become the subject of increasing debate. This discussion is likely to intensify following the publication of the combined PANORAMIC (United Kingdom) and CanTreatCOVID (Canada) trials in the *New England Journal of Medicine*. In these two open-label randomized trials, nirmatrelvir–ritonavir did not significantly reduce the incidence of hospitalization or death among vaccinated, higher-risk outpatients with SARS-CoV-2 infection [9,10].

Molnupiravir has not received centralized marketing authorization from the European Medicines Agency (EMA). Nevertheless, the World Health Organization continues to issue a conditional recommendation for its use in selected high-risk patients with non-severe COVID-19, while emphasizing the limited safety data and the need for careful patient selection and pharmacovigilance. Concerns regarding potential genotoxicity and reproductive toxicity have contributed to restrictions on its use, particularly in pregnant women and children [11]. Remdesivir has shown limited practicality in outpatient settings and uncertain clinical benefit in hospitalized patients, while concerns regarding adverse effects have also been reported [12].

The evidence supporting immunomodulatory therapies is similarly heterogeneous. Results for tocilizumab have been inconsistent across clinical studies. Although the large randomized RECOVERY trial demonstrated a statistically significant reduction in mortality, the absolute benefit was modest, corresponding to an absolute mortality reduction of approximately 4% [13].

## 2. Contemporary Integrated Model of Severe COVID-19

Current evidence indicates that severe COVID-19 is not the consequence of a single pathogenic event, such as an isolated “cytokine storm,” but rather represents the culmination of multiple interconnected biological processes that collectively drive disease progression. Accordingly, severe COVID-19 is increasingly conceptualized as a complex syndrome resulting from the dynamic interplay among viral cytopathic injury, immune dysregulation, endothelial dysfunction, and thromboinflammation [14,15].

Several major pathogenic pathways contribute to this integrated model. These include direct virus-mediated tissue injury, activation of pro-inflammatory cytokine networks, dysregulation of the bradykinin signaling pathway, excessive accumulation of hyaluronan within the pulmonary interstitium, endothelial injury and dysfunction, and activation of coagulation pathways leading to microvascular thrombosis and systemic coagulopathy [14,15,16,17,18,19].

Importantly, these mechanisms do not operate independently but constitute a highly interconnected biological network sustained by multiple positive feedback loops. Viral tissue injury amplifies inflammatory responses, while inflammation promotes endothelial activation and coagulation. In turn, endothelial dysfunction and microvascular thrombosis exacerbate tissue hypoxia and organ damage, further intensifying inflammatory signaling. The result is the establishment of self-perpetuating cycles of inflammation, vascular injury, and thrombosis that may persist even after the initial viral burden has begun to decline [14,20].

In susceptible individuals, particularly those with advanced age, obesity, diabetes mellitus, cardiovascular disease, or pre-existing chronic inflammatory conditions, these self-amplifying processes may lead to rapid clinical deterioration. Progressive impairment of pulmonary gas exchange can culminate in severe hypoxemia, diffuse alveolar damage, acute respiratory distress syndrome (ARDS), and ultimately respiratory failure. Furthermore, systemic endothelial injury and widespread immunothrombosis contribute to multiorgan dysfunction, a defining feature of critical COVID-19 [21].

Tissue Factor (TF) is increasingly recognized as a central molecular mediator linking NLRP3 inflammasome activation to the coagulation cascade. This signaling axis places the inflammasome at the center of contemporary models of thromboinflammation in severe COVID-19 by integrating inflammatory, endothelial, and hemostatic disturbances into a unified pathogenic framework. NLRP3 activation also promotes neutrophil extracellular trap (NET) formation through IL-1β-dependent mechanisms. NETs further amplify immunothrombosis by carrying and exposing Tissue Factor, enhancing TF expression in monocytes, and providing a structural scaffold for platelet adhesion and fibrin deposition [22,23,24].

**Figure 1:**
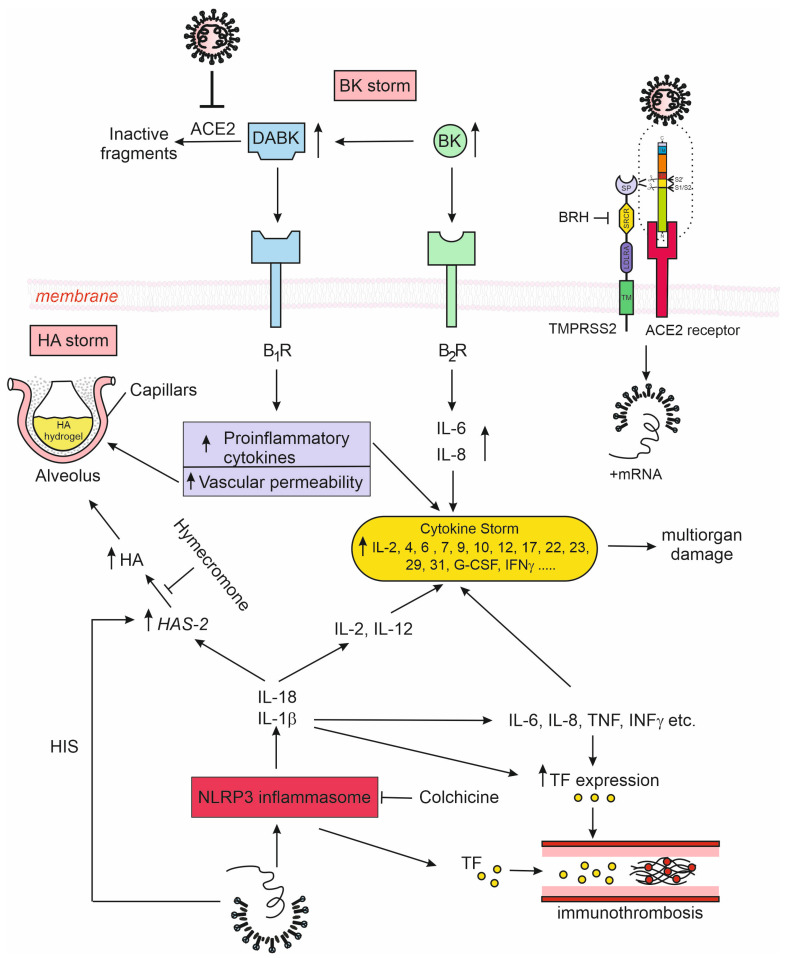
Integrated Pathophysiological Framework in Severe COVID-19: Interplay Between the NLRP3 Inflammasome, Cytokine Storm, Bradykinin Dysregulation, Hyaluronan Accumulation, and Immunothrombosis.

Taken together, contemporary concepts of severe COVID-19 support the view that disease progression results from the convergence of viral pathogenicity, dysregulated host immune responses, endothelial dysfunction, and thromboinflammatory mechanisms. This integrated pathogenic framework has important therapeutic implications, suggesting that successful management of severe COVID-19 may require simultaneous modulation of multiple interconnected pathways rather than targeting a single inflammatory mediator (Figure 1) [14,15,16,17,18,19].

Current evidence supports the concept that severe COVID-19 results from the convergence of multiple interdependent biological processes rather than from a single dominant pathogenic mechanism. Central to this integrated model is the interaction among SARS-CoV-2-induced disruption of the ACE2 axis, dysregulation of the kallikrein–kinin system, activation of the NLRP3 inflammasome, and downstream thromboinflammatory responses (Figure 1).

Following SARS-CoV-2 binding and internalization of ACE2, the physiological degradation of des-Arg^9^-bradykinin (DABK) is impaired, leading to its accumulation and the development of a so-called “bradykinin storm.” Elevated DABK levels produce sustained activation of the bradykinin B1 receptor (B1R), resulting in increased vascular permeability and enhanced production of pro-inflammatory cytokines. The consequent endothelial hyperpermeability facilitates inflammatory cell infiltration while promoting plasma leakage into the pulmonary interstitium [16,25].

Bradykinin-mediated endothelial dysfunction acts synergistically with NLRP3 inflammasome activation. It not only potentiates inflammasome-driven cytokine responses but also facilitates the extravasation of plasma constituents, including hyaluronan precursors, into the alveolar compartment as a consequence of increased capillary permeability. Simultaneously, NLRP3 activation promotes the maturation and release of IL-1β and IL-18, which upregulate the expression of hyaluronan synthase-2 (HAS2). This, in turn, drives excessive synthesis and accumulation of hyaluronan within the pulmonary interstitium and alveolar spaces, a phenomenon increasingly described as a “hyaluronan storm.” Owing to its highly hydrophilic properties, hyaluronan retains large amounts of water, thereby amplifying pulmonary edema, impairing gas diffusion, and aggravating hypoxemia [15,19].

These interconnected mechanisms establish a self-reinforcing pathogenic network in which bradykinin-mediated vascular leakage, inflammasome-driven hyperinflammation, and hyaluronan accumulation reciprocally amplify one another. This network is further integrated into the broader framework of COVID-19-associated thromboinflammation, in which NLRP3 activation promotes endothelial dysfunction, Tissue Factor expression, neutrophil extracellular trap (NET) formation, and immunothrombosis [20,24,26].

Within this integrated framework, SARS-CoV-2-induced ACE2 dysregulation initiates activation of the kallikrein–kinin system while simultaneously priming inflammatory and coagulation pathways through NLRP3 inflammasome activation. The convergence of these processes culminates in progressive endothelial barrier disruption, alveolar flooding, microvascular thrombosis, impaired pulmonary gas exchange, and ultimately severe respiratory failure.

Collectively, severe COVID-19 can be conceptualized as a multi-axis pathogenic syndrome driven by the coordinated dysregulation of three tightly interconnected biological systems:

- The bradykinin axis, promoting vascular permeability and pulmonary edema;
- The NLRP3 inflammasome axis, driving cytokine amplification, pyroptosis, and induction of hyaluronan synthase-2 (HAS2);
- The immunothrombotic axis, characterized by endothelial injury, Tissue Factor activation, NET formation, and microvascular thrombosis.

The convergence of these three pathogenic axes provides a unified mechanistic framework for the rapid progression to severe hypoxemia, acute respiratory distress syndrome (ARDS), and multiorgan dysfunction that characterizes critical COVID-19.

## 3. Overview of the NLRP3 Inflammasome

Inflammasomes are cytosolic multiprotein complexes of the innate immune system that assemble in response to pathogen-associated molecular patterns (PAMPs) and danger-associated molecular patterns (DAMPs). Their activation leads to the recruitment and activation of caspase-1, which mediates the proteolytic maturation and secretion of the pro-inflammatory cytokines interleukin (IL)-1β and IL-18, as well as the cleavage of gasdermin D, thereby inducing pyroptotic cell death. By coordinating inflammatory responses to microbial infection and sterile cellular stress, inflammasomes play a central role in host defense, tissue homeostasis, and immune regulation. Conversely, dysregulated inflammasome activation contributes to the pathogenesis of numerous inflammatory, autoimmune, metabolic, cardiovascular, and neurodegenerative disorders, making these complexes attractive therapeutic targets. Among the currently identified inflammasomes, NLRP3 is by far the most extensively investigated because of its ability to respond to an exceptionally broad spectrum of infectious and sterile stimuli [27,28].

The NLRP3 inflammasome is a central effector of innate immune signaling and consists of three core components: the sensor protein NLRP3, the adaptor protein apoptosis-associated speck-like protein containing a caspase recruitment domain (ASC), and the effector zymogen pro-caspase-1. Structurally, NLRP3 comprises three functional domains: a C-terminal leucine-rich repeat (LRR) domain involved in sensing cellular stress signals, a central NACHT domain responsible for ATP-dependent oligomerization, and an N-terminal pyrin (PYD) domain that mediates protein–protein interactions required for inflammasome assembly. This modular organization enables NLRP3 to function as a molecular switch, remaining inactive under physiological conditions while rapidly responding to diverse danger signals. Although NLRP3 activation has been studied predominantly in myeloid cells, its expression has also been demonstrated in a variety of non-myeloid cell types, including bronchial epithelial cells and lung fibroblasts, highlighting its broader role in tissue homeostasis and inflammatory disease [29,30,31].

Given its pivotal role in numerous pathological processes, NLRP3 inflammasome activity is subject to exceptionally complex and tightly coordinated regulation. Multiple layers of control govern its activation, including protein interactors; microRNAs (miRNAs); long non-coding RNAs (lncRNAs); post-translational modifications such as phosphorylation, ubiquitination, SUMOylation, S-nitrosylation, ADP-ribosylation, acetylation, O-GlcNAcylation, Tyr861 nitration, glycosylation, and palmitoylation; as well as metabolites, lipids, ions, and diverse small-molecule modulators. Based on the literature available through 2025–2026, more than 300 individual molecules have been reported to directly or indirectly regulate NLRP3 inflammasome activation or function, underscoring the remarkable complexity of the molecular networks governing this signaling platform [31,32].

## 4. The Central Role of the NLRP3 Inflammasome in the Shared Pathogenic Mechanisms of COVID-19 and Influenza

### 4.1. The NLRP3 Inflammasome: Common Ground Despite the Differences Between COVID-19 and Influenza

The NLRP3 inflammasome plays a pivotal role in the pathogenesis of both COVID-19 and influenza by promoting excessive inflammatory responses through activation of caspase-1 and the subsequent maturation and release of IL-1β and IL-18. Dysregulated NLRP3 activation contributes to lung injury, cytokine dysregulation, endothelial dysfunction, immunothrombosis, disease severity, and adverse clinical outcomes in both viral infections [33,34,35,36,37].

COVID-19 and influenza share several fundamental pathogenic mechanisms despite being caused by distinct viruses. Both pathogens rely on host protease-mediated activation for efficient viral entry, with TMPRSS2 playing an important role in the activation of viral surface glycoproteins. In both infections, severe disease is characterized by dysregulated activation of the NLRP3 inflammasome, resulting in excessive IL-1β-driven inflammation, pulmonary tissue injury, and progressive respiratory dysfunction. These shared mechanisms identify the NLRP3 inflammasome as a common upstream pathogenic axis and a potential therapeutic target [31,32,33,34,35,36,37,38,39].

The mechanistic rationale for targeting NLRP3 is supported by the substantial overlap in the pathogenic pathways underlying severe COVID-19 and influenza. Although the two diseases differ in virology and tissue tropism, their severe clinical manifestations converge on common inflammatory and thromboinflammatory processes, with NLRP3 inflammasome hyperactivation representing a central upstream event. Increasing evidence indicates that disease severity is determined not only by viral replication but also by an exaggerated and dysregulated host immune response [26,40,41,42].

### 4.2. Therapeutic Implications of Targeting the NLRP3 Inflammasome with Colchicine

The purpose of this review is to summarize the mechanistic rationale and available evidence supporting pharmacological modulation of the NLRP3 inflammasome as a strategy for preventing severe COVID-19 and influenza, with particular emphasis on colchicine-based approaches.

The integrated model suggests that early modulation of NLRP3 activity may attenuate multiple downstream pathogenic processes simultaneously. In contrast, antiviral therapies primarily suppress viral replication without directly targeting inflammasome activation, while therapeutic strategies directed against individual downstream mediators, such as IL-6, may provide only partial control of inflammation if persistent upstream NLRP3 activation continues to drive cytokine production and thromboinflammatory responses [10,15,31,43,44,45,46,47,48,49].

Taken together, these observations suggest that early pharmacological modulation of excessive NLRP3 inflammasome activation may represent an important strategy for preventing progression to severe COVID-19 and influenza.

Although numerous compounds have demonstrated inhibitory effects on NLRP3 activation in experimental models, only a limited number have shown favorable efficacy and safety profiles in vivo, and no selective direct NLRP3 inhibitor has yet received regulatory approval for clinical use [32]. Among the most promising repurposed agents is colchicine, a well-established anti-inflammatory drug currently approved for the treatment of acute gout, familial Mediterranean fever, Behçet’s disease, recurrent pericarditis, and several other inflammatory disorders [50,51].

Colchicine inhibits NLRP3 inflammasome activation at micromolar concentrations in vitro and possesses pharmacokinetic characteristics that distinguish it from many experimental NLRP3 inhibitors. Notably, colchicine preferentially accumulates within myeloid cells, where intracellular concentrations may substantially exceed those measured in plasma. Because myeloid cells are major mediators of hyperinflammation and express high levels of NLRP3, preferential intracellular accumulation may permit pharmacologically relevant modulation of inflammasome activity despite relatively low circulating drug concentrations. In addition, emerging evidence suggests that colchicine may exert antiviral effects while modulating inflammatory responses without producing broad immunosuppression [47,48].

### 4.3. The Therapeutic Rationale for Colchicine in COVID-19

The therapeutic rationale for colchicine in COVID-19 is closely linked to the disease’s prominent thrombo-inflammatory phenotype. SARS-CoV-2 infection induces endothelial dysfunction, platelet activation, NET formation, and excessive activation of the NLRP3 inflammasome, which together promote vascular inflammation and immunothrombosis. By disrupting microtubule polymerization, colchicine indirectly suppresses NLRP3 inflammasome activation, inhibits neutrophil chemotaxis and activation, reduces inflammasome-dependent cytokine release, and attenuates several upstream mechanisms contributing to endothelial injury and thrombo-inflammation. Consequently, colchicine may exert beneficial effects not only through modulation of systemic inflammation but also by limiting the vascular complications that characterize severe COVID-19 [52].

### 4.4. The Therapeutic Rationale for Colchicine in Influenza

In influenza, the inflammatory response differs from that observed in COVID-19, with a greater emphasis on epithelial injury, innate immune activation, and cytokine-mediated pulmonary inflammation. Although NLRP3 inflammasome activation also contributes to influenza pathogenesis, endothelial dysfunction, systemic vascular inflammation, and immunothrombosis generally play a less prominent role than in severe COVID-19. Therefore, the potential therapeutic effects of colchicine in influenza are thought to derive primarily from suppression of excessive neutrophil recruitment, indirect inhibition of NLRP3 inflammasome activation, and reduction in pro-inflammatory cytokine production rather than from modulation of thrombo-inflammatory pathways [53].

### 4.5. Indirect Inhibition of the NLRP3 Inflammasome by Colchicine

Despite these pathophysiological differences, NLRP3 inflammasome activation represents a common therapeutic target in both diseases. Recent structural studies, including cryo-electron microscopy analyses, have provided important insights into the architecture of the NLRP3 inflammasome and the binding modes of direct NLRP3 inhibitors [54].

Unlike selective NLRP3 small-molecule inhibitors that directly target the NLRP3 protein and inhibit inflammasome activation through specific molecular interactions, colchicine is not considered a direct NLRP3 inhibitor. Instead, colchicine exerts its anti-inflammatory effects primarily through binding to tubulin and inhibition of microtubule polymerization. This disruption of microtubule dynamics affects intracellular trafficking, mitochondrial positioning and function, and cellular events required for efficient inflammasome assembly. Consequently, colchicine reduces NLRP3 activation by interfering with upstream regulatory processes, including ASC recruitment and speck formation, leading to decreased caspase-1 activation and subsequent maturation of IL-1β and IL-18. Therefore, colchicine should be regarded as an indirect modulator of NLRP3 inflammasome activation through cytoskeletal regulation rather than as a direct molecular inhibitor of NLRP3 [55,56].

### 4.6. Direct Inhibition of the NLRP3 Inflammasome

In contrast to colchicine, several selective direct NLRP3 inhibitors have been developed to specifically target the NLRP3 inflammasome and are currently undergoing clinical evaluation. These compounds, including dapansutrile (OLT1177), inzomelid (IZD174), selnoflast (IZD334), and ZYIL1, act through direct inhibition of NLRP3 activation and have been investigated in inflammatory conditions such as gout, cryopyrin-associated periodic syndromes (CAPS), cardiovascular diseases, and other immune-mediated disorders. Structural and biochemical studies have provided evidence supporting direct interaction of some of these small molecules with the NLRP3 inflammasome complex, particularly through modulation of the NLRP3 NACHT domain or associated regulatory mechanisms. Among these agents, dapansutrile (OLT1177) is currently the most advanced clinical candidate, having progressed to Phase II/III clinical trials for selected indications, whereas most other direct NLRP3 inhibitors remain in Phase I or II development [57]. The emergence of selective direct NLRP3 inflammasome inhibitors may further improve targeted anti-inflammatory therapy.

Nevertheless, even with the anticipated approval of selective direct NLRP3 inflammasome inhibitors, colchicine is expected to remain an important therapeutic option because of its markedly lower cost, global availability, and well-established clinical use.

Collectively, these observations support the concept that severe COVID-19 and influenza converge on a common upstream pathogenic pathway centered on NLRP3 inflammasome hyperactivation. This provides a strong biological rationale for investigating therapeutic strategies aimed at modulating NLRP3 activity in order to interrupt the cascade leading to hyperinflammation, endothelial injury, and thromboinflammatory complications [31].

## 5. Therapeutic Implications of Targeting the NLRP3 Inflammasome

**Our proposed high-dose colchicine regimen**

Initially, we employed a weight-adjusted loading regimen based on the formula 0.5 mg of colchicine per 10 kg body weight minus 0.5 mg, with a maximum loading dose of 5 mg during the first 24 h. According to this regimen, the maximal loading dose is reached in individuals weighing approximately 110 kg, while patients with higher body weight receive the same maximum dose. This is followed by a maintenance regimen corresponding to half of the loading dose, resulting in a cumulative colchicine dose of 15 mg over 5 days [15,58,59,60,61,62].

Other investigators have used substantially different dosing protocols, including 2 mg/day (cumulative dose of 10 mg over 5 days) [63,64], 4 mg/day (cumulative dose of 20 mg over 5 days) [63], or a tapering regimen of 4–3.5–3–2.5–2 mg (cumulative dose of 15 mg over 5 days) [51,63]. Available evidence suggests a statistically significant clinical benefit of a cumulative dose of 20 mg compared with 10 mg over the same treatment period [63,64].

In critically ill patients with an immediate life-threatening condition, we have occasionally administered a loading dose of 6 mg within the first 24 h [51]. However, tolerance to higher colchicine doses varies considerably among individuals, and dose escalation should therefore be guided by the patient’s clinical response and tolerability.

Although the currently available evidence remains limited, it suggests that a minimum cumulative colchicine dose of 10 mg over 5 days may be required to achieve sufficient suppression of the hyperinflammatory response and cytokine storm. Further well-designed prospective studies are needed to define the optimal colchicine dosing regimen, with particular emphasis on the interaction between dose, cumulative exposure, and the timing of treatment initiation.

## 6. Clinical Observations Following High-Dose Colchicine in Severe and Critical COVID-19

### 6.1. Case Series

During the early phase of the COVID-19 pandemic, when mortality was high and effective therapeutic options were limited, we hypothesized that administering high loading doses of colchicine—comparable to those previously evaluated in randomized clinical trials—might achieve intracellular drug concentrations within myeloid cells sufficient to inhibit NLRP3 inflammasome activation. Because colchicine preferentially accumulates in leukocytes, this strategy was intended to attenuate the excessive inflammatory response underlying severe COVID-19. The favorable clinical responses observed prompted further clinical application of this regimen, and several representative cases were subsequently published.

Our published case series describes several patients with severe or critical COVID-19 who experienced rapid clinical improvement following administration of high-dose colchicine. Although these observations cannot establish causality, they provide hypothesis-generating clinical evidence supporting further investigation of this therapeutic approach.

One representative case involved a patient with extensive bilateral COVID-19 pneumonia and acute respiratory distress syndrome (ARDS). Chest computed tomography demonstrated diffuse pulmonary involvement, with only approximately 10% of the lung parenchyma remaining unaffected. Despite the extremely poor prognosis, the patient recovered following treatment with high-dose colchicine [15].

Patients with severe obesity (body mass index [BMI] > 40 kg/m^2^) represent one of the highest-risk populations for progression to critical COVID-19. One patient weighing 120 kg, with morbid obesity, hypertension, type 2 diabetes mellitus, and gout, was admitted to hospital on the third day after diagnosis with an oxygen saturation of 89%. Despite standard treatment, his respiratory status progressively deteriorated, with oxygen saturation declining to 74%. Administration of a 6 mg loading dose of colchicine on the eighth day of illness was followed by rapid clinical improvement and subsequent recovery [51].

A second patient, a 34-year-old man weighing 184 kg with insulin resistance, was managed as an outpatient until day 7 of illness, when he developed the abrupt respiratory deterioration characteristic of severe COVID-19. Following consultation with our group, he received a 5 mg loading dose of colchicine. High fever and severe myalgia resolved rapidly after treatment, followed by progressive clinical recovery. Two additional patients with morbid obesity (BMI > 50 and >60 kg/m^2^) likewise demonstrated rapid clinical improvement after receiving a single 5 mg loading dose of colchicine [58].

Another notable case involved a 101-year-old man weighing 70 kg who was recovering in the intensive care unit after two surgical procedures. Following nosocomial SARS-CoV-2 infection, he received colchicine immediately at a dose of 4 mg (0.06 mg/kg) and subsequently recovered despite his exceptionally high-risk clinical profile [59].

Collectively, these cases illustrate favorable clinical outcomes across diverse high-risk patient populations, including individuals with advanced age, morbid obesity, multiple comorbidities, and severe pulmonary involvement.

An additional illustrative observation involved a married couple who both developed bilateral COVID-19 pneumonia during the same period. The two patients were admitted to different hospitals employing different therapeutic protocols. The wife was treated in a center where high-dose colchicine formed part of the institutional treatment strategy and recovered, whereas her husband, treated at another institution without colchicine, died from COVID-19 [60]. Although this anecdotal observation does not permit conclusions regarding treatment efficacy, it illustrates the marked differences in clinical outcomes that may occur under different management approaches.

Interestingly, isolated reports have described favorable outcomes following inadvertent colchicine overdoses (12.5–15 mg), including complete resolution of COVID-19-associated pericardial effusion [60,61]. Although such observations should be interpreted with considerable caution because of the well-recognized toxicity of colchicine at high doses, they raise the hypothesis that sufficiently high intracellular drug exposure may produce biological effects distinct from those achieved with conventional dosing regimens.

Overall, these clinical observations should be regarded as exploratory and hypothesis-generating rather than confirmatory. Nevertheless, together with the growing mechanistic understanding of NLRP3 inflammasome biology, they provide additional justification for rigorous evaluation of optimized colchicine dosing strategies in adequately powered randomized clinical trials.

### 6.2. Clinical Evidence for High-Dose Colchicine in Outpatient and Hospital Settings

To further evaluate the potential clinical effects of high-dose colchicine in patients with COVID-19, we conducted a multicenter observational study involving both hospitalized and outpatient populations. Rather than a randomized controlled design, the study employed a historical comparison, evaluating clinical outcomes before and after the introduction of high-dose colchicine into routine practice. Although this design cannot eliminate the influence of temporal or other confounding factors, it provided an opportunity to assess treatment outcomes under real-world clinical conditions.

#### 6.2.1. Hospitalized Patients

The inpatient cohort included 795 patients treated in four hospitals in Bulgaria. Before implementation of the high-dose colchicine protocol, hospital mortality was comparable to the national average among hospitalized patients with COVID-19. Following introduction of the protocol, patients received cumulative colchicine doses ranging from 10 to 20 mg over five days.

Implementation of high-dose colchicine was associated with a substantial reduction in in-hospital mortality, with the magnitude of benefit increasing across higher cumulative dose categories. Furthermore, cumulative colchicine dose demonstrated a statistically significant inverse association with mortality [62,63].

An important limitation affecting treatment outcomes was the timing of hospital admission. Patients who died despite colchicine treatment generally presented considerably later in the course of illness than those who recovered, emphasizing the importance of initiating therapy before the development of advanced hyperinflammation. These observations are consistent with the hypothesis that early modulation of NLRP3 inflammasome activation may be more effective than intervention after extensive cytokine amplification and thromboinflammatory injury have become established. Favorable outcomes were observed across patients with advanced age, obesity, diabetes mellitus, and multiple comorbidities, although the observational nature of the study precludes definitive conclusions regarding treatment efficacy within specific risk groups [51,58,59].

#### 6.2.2. Outpatient Observations

Our outpatient analyses likewise demonstrated favorable clinical associations with high-dose colchicine treatment. Increasing cumulative colchicine exposure was associated with a lower risk of hospitalization, while longer treatment duration correlated with reduced hospitalization rates and a lower frequency of persistent post-COVID-19 symptoms [60].

From a mechanistic perspective, colchicine differs from currently recommended antiviral therapies, including nirmatrelvir/ritonavir, remdesivir, and molnupiravir. Whereas antiviral agents primarily inhibit viral replication, colchicine targets host inflammatory pathways by modulating NLRP3 inflammasome activation and downstream thromboinflammatory responses. Consequently, its potential therapeutic effects may extend beyond the antiviral phase of disease and remain relevant during both outpatient management and hospitalization.

Colchicine also offers several practical advantages. It has a well-established safety profile when used appropriately, predictable and generally manageable adverse effects, low cost, and worldwide availability, characteristics that make it an attractive candidate for repurposing, particularly in resource-limited healthcare settings [49].

### 6.3. Comparison with Subsequent Clinical Studies

Subsequent evidence has provided additional support for evaluating higher-dose colchicine regimens. A randomized, double-blind clinical trial reported that a cumulative colchicine dose of 10 mg administered over five days was associated with significantly lower mortality than standard care (6.6% versus 25.0%, *p* = 0.006) [64].

Similarly, in our multicenter observational study, a cumulative five-day dose of 9.5 mg was associated with a statistically significant reduction in mortality (relative risk 0.423; 95% confidence interval 0.253–0.707; *p* = 0.001), corresponding to an estimated relative risk reduction of 57.7% [63].

Additional clinical studies employing cumulative five-day colchicine doses between 6 and 7.5 mg have also reported favorable outcomes, including reduced mortality, delayed clinical deterioration, shorter duration of supplemental oxygen therapy and hospitalization, reduced intensive care unit stay, decreased need for mechanical ventilation, and more rapid clinical recovery [65,66,67,68].

Taken together, the currently available observational studies and randomized clinical data suggest that colchicine dosing may represent an important determinant of therapeutic efficacy. Although further adequately powered randomized controlled trials are required to define the optimal treatment regimen, the existing evidence supports continued investigation of higher-dose colchicine strategies for patients with COVID-19.

## 7. The World Health Organization Living Guideline Recommends Against Colchicine for COVID-19: Is Reappraisal Warranted?

The current World Health Organization (WHO) living guideline recommends against the use of colchicine for the treatment of COVID-19, primarily on the basis of evidence derived from large randomized controlled trials (RCTs) and subsequent meta-analyses [68,69,70]. Overall, the available clinical evidence has been inconsistent, with individual trials reporting both positive and negative findings [70].

A major limitation in interpreting the existing RCTs and their pooled analyses is the substantial heterogeneity among studies. Critical determinants of colchicine efficacy, such as the interval from symptom onset to treatment initiation, body weight (which prevents accurate assessment of dose per kilogram), age, comorbidities, disease severity, and concomitant therapies, are often inadequately reported or vary considerably across studies. This variability likely contributes to the inconsistent clinical outcomes observed among trials and limits the reliability of conventional meta-analyses. Furthermore, the absence of unified and standardized endpoint definitions across clinical studies, including hospitalization rate, mortality rate, and duration of oxygen therapy, represents an additional source of heterogeneity in the outcomes reported by meta-analyses [47,64].

It should be emphasized that the favorable outcomes reported with high-dose colchicine regimens apply only to carefully selected patients without significant hepatic or renal impairment and in the absence of clinically relevant drug–drug interactions [47].

**Figure 2:**
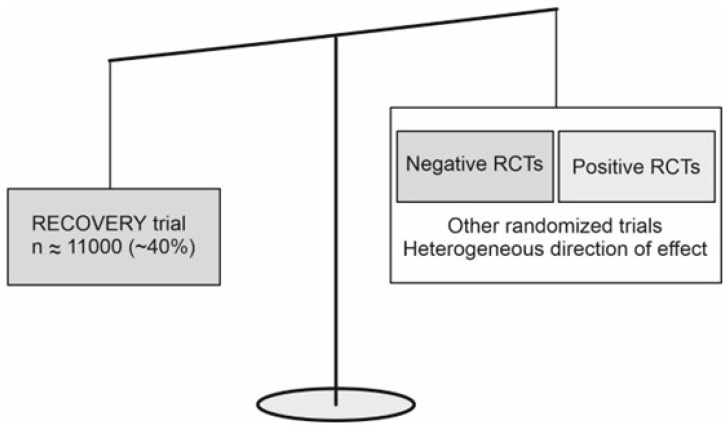
Contribution of the RECOVERY Trial to the Randomized Evidence Base Evaluating Colchicine for COVID-19.

Among these studies, the RECOVERY trial, the largest randomized clinical trial evaluating colchicine in hospitalized patients with COVID-19, has had the greatest influence on the overall interpretation of the evidence and, consequently, on subsequent meta-analyses and international treatment recommendations (Figure 2) [69,70,71,72].

However, an important question remains insufficiently explored. Do the predominantly negative findings indicate that colchicine is intrinsically ineffective for COVID-19, or could they instead reflect limitations related to the timing of treatment initiation, dose selection, treatment duration, patient selection, or other aspects of trial design?

Addressing this question is essential because the biological activity of colchicine is closely linked to intracellular drug accumulation and modulation of host inflammatory pathways rather than direct antiviral effects. Consequently, therapeutic efficacy may depend critically on whether treatment is initiated before irreversible hyperinflammation and thromboinflammatory injury become established. The following sections therefore examine the principal randomized clinical trials of colchicine in COVID-19 with particular emphasis on dosing strategies, timing of administration, and their relationship to the current understanding of NLRP3 inflammasome biology.

The RECOVERY trial enrolled approximately 40% of all participants included in pooled randomized analyses of colchicine for COVID-19 (11,340 of 28,249 participants) [69,70]. Owing to its substantially larger sample size, RECOVERY exerts considerable statistical influence on pooled effect estimates and has therefore had a major impact on subsequent meta-analyses and the development of clinical practice guidelines. The remaining evidence base comprises multiple smaller randomized controlled trials that reported heterogeneous findings.

Schematic illustration. The relative sizes of the graphical elements are proportional to study sample size. Colors indicate the overall direction of the reported treatment effect (favorable, neutral, or unfavorable) and are intended for illustrative purposes only; they do not represent the magnitude of treatment effect or statistical significance.

As we have previously discussed, the interpretation of the RECOVERY trial requires careful consideration of the specific treatment regimen that was evaluated. The authors concluded that *“To date there has been no convincing evidence of the effect of colchicine on clinical outcomes in patients admitted to hospital with COVID-19”* [70]. However, this conclusion should be interpreted within the context of the trial design, which evaluated colchicine initiated in hospitalized patients using a relatively low-dose regimen rather than early intervention or higher-dose treatment strategies [47].

Our findings do not challenge the internal validity of the RECOVERY trial but instead address a different therapeutic hypothesis. The RECOVERY trial provides strong evidence that low-dose colchicine initiated after hospitalization does not improve clinical outcomes in COVID-19. In contrast, our observational studies and the emerging evidence from higher-dose clinical trials suggest that the timing of treatment initiation and cumulative colchicine exposure may be critical determinants of therapeutic efficacy [63,64]. Specifically, early administration before the onset of advanced hyperinflammation, together with higher cumulative dosing, may produce substantially different clinical outcomes than the regimen evaluated in RECOVERY. This hypothesis warrants prospective evaluation in adequately powered randomized clinical trials.

## 8. Colchicine Toxicity in Clinical Practice

The safety profile of colchicine at higher doses is of considerable clinical interest in light of emerging evidence suggesting potential therapeutic benefits of optimized dosing strategies in severe COVID-19 [47,64] and, potentially, influenza [31]. Accurate assessment of colchicine toxicity therefore requires careful interpretation of the available clinical evidence, including recognition of factors that may substantially modify toxicity risk.

Several recent analyses have emphasized that the risk of severe colchicine toxicity is strongly influenced by patient-specific factors, particularly clinically significant drug–drug interactions and underlying hepatic or renal dysfunction [71,72]. Accordingly, interpretation of older reports describing fatal intoxication should consider whether these important predisposing factors were present. For example, cases involving reported fatal doses of 7–7.5 mg, originally described by Finkelstein and colleagues, were subsequently re-evaluated and found to involve major contributing factors, including severe pharmacokinetic drug interactions and hepatic impairment, rather than colchicine exposure alone [47,71,73,74].

A systematic review of published case reports spanning the period from 1947 to 2025 (Table 1) suggests that, after excluding patients with clinically significant drug–drug interactions or substantial hepatic or renal impairment, no fatal colchicine intoxications have been documented at doses below 0.2 mg/kg [71,72]. Within the available literature, doses below 0.1 mg/kg have generally been well tolerated, whereas exposures between 0.1 and 0.2 mg/kg have occasionally been associated with toxicity but, in the absence of major predisposing factors, have not been linked to fatal outcomes [71,72].

These observations should be interpreted cautiously, as they are derived primarily from published case reports and observational evidence rather than prospective dose-escalation studies. Nevertheless, they suggest that patient characteristics, comorbidities, organ function, and concomitant medications are likely to be major determinants of colchicine toxicity and should be carefully considered when evaluating the safety of higher-dose treatment strategies.

For example, reported fatalities at approximately 0.17 mg/kg have occurred in patients with significant predisposing factors, including chronic kidney disease [81] or concomitant administration of cyclosporine, resulting in a clinically relevant drug–drug interaction [84].

One reported fatal case involving ingestion of 5 mg of colchicine was attributed by the authors to “delayed elimination and accumulation.” The patient, a 70-year-old woman, died after 12 days of intensive care, with a postmortem blood colchicine concentration of 5 ng/mL. While the prolonged elimination profile is suggestive of impaired drug clearance, potentially related to hepatic and/or renal dysfunction, the report does not provide information regarding body weight, comorbidities, organ function, or concomitant medications. These missing data preclude a full assessment of contributing risk factors that may have influenced the outcome [82].

An acute overdose of 18 mg (approximately 0.25 mg/kg) has been reported to result in multiorgan failure and death, with an antemortem blood colchicine concentration of 14 ng/mL measured 18.5 h after ingestion [85]. Consistent with pharmacokinetic data, clinically significant toxicity is generally associated with serum colchicine concentrations exceeding 3.0 ng/mL [86].

Another published conference report describes a fatal outcome following ingestion of 10 mg of colchicine over approximately one hour in a patient with severe hepatic impairment and unspecified comorbidities [87]. In the absence of information on body weight and detailed clinical characteristics, the relationship between the ingested dose and the fatal outcome cannot be reliably interpreted.

Similarly, a brief report describes the death of a 37-year-old woman following ingestion of 15 mg of colchicine [88]. However, limited clinical information is available, including data on body weight, concomitant medications, alcohol use, and hepatic or renal function, which restricts interpretation of potential contributing factors.

The frequently cited case of fatal colchicine toxicity following ingestion of 18 mg (approximately 0.2 mg/kg) in a 48-year-old patient was also associated with relevant predisposing factors, including hepatic impairment and possible drug–drug interactions, which may have contributed to the clinical course [89].

Another fatal case involved a 16-year-old girl of unknown body weight who ingested 12.5 mg of colchicine in combination with five additional medications, making it difficult to disentangle the potential contribution of concomitant drug exposure to the outcome [90].

In several published reports, body weight is not provided, preventing calculation of weight-adjusted exposure. This limitation is important, as absolute dose alone may be misleading. For instance, ingestion of 11 mg in a patient of unknown body weight does not allow reliable estimation of mg/kg exposure [91]. Conversely, in another fatal case involving a 15-year-old boy, ingestion of 18 mg corresponded to an estimated dose of approximately 0.4 mg/kg, illustrating the importance of weight-adjusted dosing in toxicological interpretation [92].

Overall, these reports highlight the substantial heterogeneity in clinical context, data completeness, and predisposing risk factors across published cases of colchicine toxicity. This variability limits direct dose–outcome comparisons based on absolute ingested amounts alone.

Detailed information regarding concomitant medications is frequently unavailable, particularly in reports of intentional overdose associated with suicide attempts. Similarly, hepatic and renal function, as well as relevant comorbidities, are often inadequately documented. Notably, in nearly all reported fatalities following relatively low colchicine doses, clinically significant drug–drug interactions, pre-existing hepatic and/or renal impairment, or both, were identified or strongly suspected as important contributing factors.

In a substantial proportion of published cases, the ingested colchicine dose is not reported [77,78,83]. In addition, case descriptions are often limited in scope and omit clinically important variables such as body weight, comorbidities, concomitant medications, and organ function. This limitation is particularly relevant in reports of intentional self-poisoning, in which co-ingestion of other substances, including alcohol and other medications, is frequently documented incompletely or not at all, thereby limiting reliable interpretation of the clinical course [77,82,87,88,93,94].

Historical reports have also contributed to widely cited assumptions regarding colchicine toxicity thresholds. In “Colchicine overdose: the devil is in the detail”, Jayaprakash et al. [77] discussed early regulatory recommendations that were largely based on the 1947 report “Hypersensitivity to colchicine” [73]. However, careful examination of the original article reveals a critical “detail”: the patient had been receiving mercury concurrently with colchicine. The authors attributed the adverse outcome to an “increased sensitivity to colchicine,” but the potential contribution of concomitant mercury exposure cannot be excluded. The claim of “increased sensitivity to colchicine” proposed by Macleod and Phillips [73] was challenged as early as 1979 in a report describing death after ingestion of 7.5 mg of colchicine (0.125 mg/kg) [95]. In that report, however, another important “detail” emerges: the 41-year-old woman had been chronically taking the veterinary drug phenylbutazone, which is capable of causing hepatic and renal injury and has been prohibited for human use in the United States since 2003. In addition, she had consumed “five or six glasses of vodka.” These two classic reports therefore represent typical examples of drug–drug interactions and underlying organ dysfunction and should not be cited as evidence for the lowest toxic or lethal dose of colchicine [71]. Taken together, these two classic reports illustrate that many of the historical cases underpinning current perceptions of colchicine toxicity involved concomitant exposures or conditions capable of substantially modifying colchicine pharmacokinetics and toxicity. Consequently, they should not be regarded as reliable evidence for defining the lowest toxic or lethal dose of colchicine.

Across the published literature, it remains difficult to identify well-documented cases of fatal colchicine poisoning at doses below 0.2 mg/kg in the absence of clinically significant drug–drug interactions or pre-existing hepatic or renal impairment. This observation is consistent with available pediatric data, in which fatal outcomes below this threshold are not well substantiated [72].

Finkelstein et al. state that “the lowest reported lethal doses of oral colchicine are 7–26 mg” [74]. However, this work does not present an original patient cohort and does not constitute a systematic quantitative analysis of individual cases. Rather, it represents a narrative synthesis of heterogeneous reports with variable methodological quality, many of which lack essential clinical information, including comorbidities, concomitant medications, and other determinants of toxicity.

Taken together, the available evidence suggests that colchicine toxicity is strongly influenced by patient-specific and context-specific factors, particularly drug–drug interactions and organ dysfunction. In this context, cumulative doses below 0.1 mg/kg are generally reported to be well tolerated in the absence of major risk factors, whereas doses between 0.1 and 0.2 mg/kg may be associated with toxicity in selected cases but have not been convincingly linked to fatal outcomes [71,72,96].

The preference for low-dose colchicine in current WHO recommendations and other clinical guidelines appears to be influenced by two main considerations. First, there has been a longstanding perception that colchicine doses in the range of 7–15 mg are inherently associated with severe toxicity, often without full consideration of the contribution of drug–drug interactions and underlying hepatic or renal impairment to reported fatal outcomes. Second, a randomized trial comparing low- and high-dose colchicine for acute gout demonstrated no significant difference in the primary clinical endpoint between dosing strategies, thereby supporting the use of lower doses in that clinical context [97].

However, as discussed above, available evidence suggests that cumulative colchicine doses below 0.1 mg/kg are generally well tolerated in the absence of major contraindications. Importantly, the pathophysiology of acute gout cannot be directly extrapolated to COVID-19, which is characterized by systemic endothelial dysfunction, thromboinflammation, and dysregulated innate immune activation. For this reason, higher-dose colchicine regimens have received limited systematic evaluation in COVID-19, while most randomized trials have focused on low-dose regimens, followed by meta-analyses yielding heterogeneous and sometimes conflicting results [47].

From a methodological perspective, future clinical trials in COVID-19 could consider evaluating a broader range of dosing strategies, including both low- and higher-cumulative-dose regimens, in order to more fully characterize potential dose–response relationships. Such an approach may be particularly relevant given the known intracellular accumulation of colchicine in myeloid cells, which are key mediators of the inflammatory response in severe COVID-19.

Gastrointestinal toxicity, particularly diarrhea, represents the most common dose-limiting adverse effect of colchicine, with its incidence increasing proportionally with cumulative drug exposure. In a multicenter, randomized, double-blind, placebo-controlled, parallel-group study, diarrhea occurred in 76.9% of patients receiving a cumulative colchicine dose of 4.8 mg over 6 h [97]. At higher cumulative doses, gastrointestinal intolerance was even more pronounced, with all patients developing diarrhea after a median of 24 h at a mean colchicine dose of 6.7 mg [98]. In light of these findings and our clinical experience, we limit the loading dose of colchicine to a maximum of 5 mg during the first 24 h (approximately 0.045 mg/kg) [15].

In the pre-COVID-19 era, the use of low-dose colchicine for acute gout flares was well supported by randomized clinical trials demonstrating similar efficacy between low- and higher-dose regimens [97]. However, extrapolation of these findings to COVID-19 is limited by substantial differences in disease pathophysiology and clinical trajectory.

Taken together, a more comprehensive dose-ranging trial design in COVID-19—incorporating multiple colchicine dosing cohorts—could potentially have provided a more detailed understanding of both efficacy and safety across different stages of disease. Such an approach would have allowed a more robust characterization of colchicine’s therapeutic window in the context of severe viral inflammation, although the optimal design of such studies remains to be established.

## 9. You Have COVID-19—What Comes Next?

At the beginning of the COVID-19 pandemic, this question was often associated with considerable uncertainty and concern. Epidemiological data indicated that approximately 80% of infected individuals experienced mild disease and recovered without hospitalization, whereas around 20% required hospital care and approximately 5% developed critical illness. Despite the use of standard-of-care therapies, including dexamethasone, anticoagulation, immunomodulatory agents such as tocilizumab, supplemental oxygen, and antibiotics for secondary bacterial infections, a proportion of patients with severe disease continued to experience poor outcomes.

A similar, although generally less severe, pattern is observed in seasonal influenza. Despite advances in supportive care and vaccination strategies, both COVID-19 and influenza continue to cause substantial morbidity and mortality globally, particularly among older adults and individuals with comorbidities such as obesity, diabetes mellitus, and cardiovascular disease.

From a mechanistic perspective, both SARS-CoV-2 and influenza viruses rely on host proteases, including TMPRSS2, for efficient cellular entry. This has led to the investigation of host-directed strategies targeting viral entry pathways. Bromhexine, a compound proposed to modulate TMPRSS2 activity, has been evaluated in both prophylactic and early therapeutic settings in limited clinical studies. Preliminary observations have suggested potential effects on clinical outcomes; however, these findings require confirmation in adequately powered randomized controlled trials [31,60,99,100,101].

In parallel, colchicine has been proposed as a host-directed anti-inflammatory agent with potential relevance to early-stage disease. Its mechanism of action involves modulation of microtubule dynamics and inhibition of NLRP3 inflammasome activation, which may be relevant to the dysregulated inflammatory response observed in severe viral infections. Experimental data also suggest possible effects on intracellular viral trafficking, although the clinical relevance of these mechanisms remains to be fully established.

Based on these mechanistic considerations, it has been hypothesized that early intervention strategies combining modulation of viral entry pathways and host inflammatory responses could influence disease progression. However, the timing of intervention, patient selection, and optimal dosing strategies require systematic evaluation in prospective clinical trials.

From a public health perspective, the development of effective, affordable, and widely accessible therapeutic options for early outpatient management of respiratory viral infections remains an important goal. Given the continuous evolution of SARS-CoV-2 and influenza viruses and the occurrence of presymptomatic transmission, host-directed therapies could, in principle, complement existing preventive and treatment strategies.

## 10. Conclusions

Hyperactivation of the NLRP3 inflammasome has emerged as a central upstream mechanism driving the excessive inflammatory response, endothelial dysfunction, immunothrombosis, and multiorgan injury that characterize severe COVID-19 and influenza. Consequently, early pharmacological modulation of this pathway, including with colchicine, represents a biologically plausible strategy to prevent disease progression.

The available mechanistic and clinical evidence suggests that the therapeutic efficacy of colchicine depends critically on both the timing of treatment initiation and cumulative drug exposure. Studies reviewed in this article indicate that higher-dose colchicine regimens, particularly when initiated early in the outpatient setting, have been associated with reduced hospitalization and lower in-hospital mortality. However, these findings should be interpreted with caution and require confirmation in adequately powered randomized controlled trials specifically designed to evaluate optimized dosing strategies and early intervention.

The toxicological evidence further indicates that colchicine safety cannot be assessed solely on the basis of the absolute ingested dose. Clinically significant drug–drug interactions, hepatic and renal dysfunction, body weight, and other patient-specific factors substantially influence the risk of toxicity. Current evidence suggests that cumulative doses below 0.1 mg/kg are generally well tolerated in patients without major contraindications, whereas doses between 0.1 and 0.2 mg/kg may cause toxicity but have not been convincingly associated with fatal outcomes in the absence of major predisposing factors. Accordingly, the widely cited concept that absolute colchicine doses of 7–7.5 mg are intrinsically lethal is not well supported by the available published evidence, as reported fatalities at these doses were generally accompanied by important confounding factors, including significant drug–drug interactions and/or hepatic or renal impairment.

Overall, the available mechanistic, clinical, and toxicological evidence supports further investigation of bromhexine as a preventive strategy for COVID-19 and influenza, as well as optimized higher-dose colchicine regimens for both outpatient and hospitalized patients. Future adequately powered randomized clinical trials are needed to define the optimal timing, dosing, patient selection, and safety profile of these therapeutic approaches.

## Figures and Tables

**Table 1:** Landmark Studies on Colchicine Poisoning Published Over the Past Eight Decades.

	Investigation	Period/Cases	Ref.
1	Stapczynski et al., 1981	1947–1979 (22 cases)	[75]
2	Bismuth et al., 1977	1966–1976 (20 cases)	[76]
3	Frankelstein et al., 2010	1966–2010 (NA)	[74]
4	Jayaprakash et al., 2007	1992–2007 (9 cases)	[77]
5	Mahmud M et al., 2025	1995–2025 (41 cases)	[78]
6	Wu and Liu, 2022	2001–2021 (20 cases)	[79]
7	Rahimie et al., 2020	2007–2014 (21 cases)	[80]
8	Fu et al., 2021	2009–2019 (43 cases)	[81]
9	Kazi et al., 2025	2011–2021 (57 cases)	[72]
10	Deng et al., 2023	2012–2023 (18 cases)	[82]
11	Seixas et al., 2021	2017–1019 (13 cases)	[83]

## Data Availability

No new data were created or analyzed in this study. Data sharing is not applicable to this article.
